# Modelling and mathematical optimisation of wastewater treatment in food industries

**DOI:** 10.12688/openreseurope.14737.1

**Published:** 2022-05-19

**Authors:** Santiago Rodriguez-Perez, Alvaro Cabeza Sanchez, María Lopez-Abelairas

**Affiliations:** 1 Biotechnology Applications, IDENER, Early Ovington 24 Nave 8-9, Seville, 41300, Spain

**Keywords:** Bioprocess modelling, multi-objective optimisation, wastewater reuse, polyhydroxyalkanoates

## Abstract

The current paper describes the work carried out in the Horizon 2020 AFTERLIFE project –  "Advanced Filtration TEchnologies for the Recovery and Later conversIon of relevant Fractions from wastEwater" – (Grant Agreement no. 745737) which focuses on bioprocess modelling and optimisation using computational tools.

The project addresses the development of a flexible, cost- and resource-efficient process framed in the zero-waste and circular economy approach for the recovery and valorisation of the relevant fractions from wastewater. The first step of such a process is an initial step consisting of a cascade of membrane filtration units to separate the total solids in sewage. Then, the concentrates recovered in each unit will be treated to obtain high-pure extracts and metabolites or to be converted into value-added biopolymers (polyhydroxyalkanoates). Moreover, the outflow of the process is an ultra-pure water stream that can be directly reused. Following a holistic approach, the design and optimisation of the AFTERLIFE process will improve performance and reduce the costs associated with wastewater treatment by maximising the value recovery.

The paper focuses on the work done developing and implementing computational tools to model and optimise the design of the process. A framework for modelling-based optimisation has been developed. The applied optimisation approach is not computationally demanding and can be systematically applied to different processes.

Finally, a use case establishing a scenario for testing the developed framework is described. The defined process model and optimisation methodology were applied to simulate the treatment of wastewater from the fish processing industry. The performance of the optimisation tool is analysed considering the simulation results.

## Plain language summary

The AFTERLIFE framework is a flexible tool for modelling and optimising processing schemes consisting of combinations of processes and/or bioprocesses. The optimisation methodology follows a multi-objective approach by combining economic and environmental criteria. It provides an optimised design of the process and indicators about its financial performance and environmental impact. Specifically, in the AFTERLIFE project, the framework has been used to simulate and optimise a wastewater treatment process consisting of an innovative scheme that allows water recovery and, simultaneously, the valorisation of the present organic matter in the production of value-added bioproducts. On the other hand, the users can easily extend the framework to simulate and optimise alternative process schemes. In this publication, a use case was described for the treatment of wastewater from a fish processing industry. The characteristics of the wastewater have been used as the input stream to the framework, and the results before and after optimisation are described. The framework is openly accessible under the Creative Commons Attribution 4.0 International License.

## Introduction

The intrinsic complexity of biological systems makes monitoring, optimisation and control challenging tasks to design a bioprocess successfully. Bioprocesses optimisation today is, in many cases, still empirical and involves either an arduous design of experiment (DoE) or availability of representative data along with data-driven methods (e.g., Artificial Neural Networks) ( Abt *et al.*, 2018). This fact makes process development time consuming and costly. Moreover, the scale-up of bioreactors and biotechnological processes is critical in industrial biotechnology. At the present stage, process development and parameters optimisation are often made at low scales, which leads to discrepant results and poorer performance in the upscaled operations. Moreover, optimisation is not addressed from a holistic perspective but at the unit level, leading to poor integration and overall performance loss.

Including mathematical models and optimisation in the workflow is a powerful tool to accelerate the optimised development and scale-up of bioprocesses, reducing the required time and resources for such tasks. Thus, efforts should be made to implement modelling-based approaches that can be broadly applicable, flexible, and robust to develop methodologies and technologies for industrial biotechnology ( Noll & Henkel, 2020). In the last decades, meaningful development and advancement of modelling techniques and a simultaneous improvement of hardware resources have occurred. Such models have been applied in different fields, including scale-up, design, optimisation, control, economic evaluation, and quality control. The modelling work in process optimisation has mainly focused on model-assisted DoE combining the inherent process knowledge of mathematical models with statistical DoE to reduce the number of required experiments and include the process dynamics ( Liang *et al.*, 2020; Luna & Martinez, 2017). Alternatively, a systematic model-based framework has been proposed by Morales-Rodriguez *et al.* (2012). Such a framework is based on stochastic programming, considering the sources of uncertainties identified through sensitivity analysis. The framework had process costs as the single optimisation objective and was developed in the commercial software Matlab. Stochastic and deterministic approaches show different advantages and drawbacks. The implementation of stochastic alternatives is typically simpler, and, in the case of optimisation algorithms, a global sampling is made, avoiding the entrapping in a local minimum. On the other hand, deterministic alternatives allow a higher control of the optimisation process. It should also be noted that the performance of the Monte-Carlo technique used for this stochastic optimisation is strongly dependent on the size of the used sample, which is often significant to reach good performance. A size of 15.000 elements was used by Morales-Rodriguez *et al.* (2012), which implies the same number of process simulations.

The current work described a novel and alternative framework for modelling-based optimisation and its application in a multistep biotechnological process. The applied optimisation approach is deterministic and not computationally demanding, and the framework can be systematically applied to different processes. It consists of using coupled mathematical process models in combination with multi-objective optimisation functions and set constraints. Open-source instruments ( *OpenModelica*, *Python*) are used for modelling and optimisation, avoiding the implication of private and costly software. Moreover, the multi-objective function allows the incorporation of different aspects (i.e., economic and environmental) in the optimisation. On the other hand, the selected process, a new treatment for the valorisation of wastewater from the food industry developed in the AFTERLIFE project, includes several operations of high interest for many bio-based industries combined in an innovative and flexible scheme. Unified mathematical models of the treatment solution have been developed through coupling the interdependent units. Such a process model was the basis for developing the optimal design by means of an optimisation problem composed of a multi-objective function and a set of related physical, environmental, and economic constraints. The model outputs and objective function before and after optimisation are compared and discussed.

## Methods

### Process scheme

**Figure 1.**
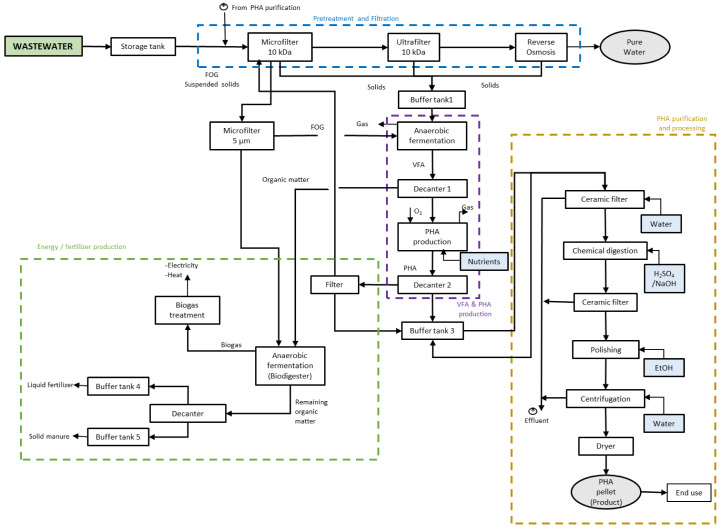
Scheme of AFTERLIFE process for the wastewater treatment.

The food processing industry is one of the main water demanding sectors in Europe, and, at the same time, it is one of the main wastewater producers. On the other hand, the generated wastewaters often contain compounds of interest (nutrients, organic matter) that could be valorised. Thus, applying a circular approach would be highly beneficial for the industries in this sector. The combination of physic and biotechnological processes has been recently proposed. The collaborative research project AFTERLIFE has developed the scheme depicted in Figure 1 for treating and valorising wastewater from food industries.

AFTERLIFE proposes a flexible, cost- and resource-efficient process framed in the zero-waste and circular economy approach to recover and valorise the relevant fractions from wastewater. The first step of such a process consists of a cascade of membrane filtration units to separate the total solids in wastewater. The low added value organic matter goes to acidogenic fermentation to produce a VFA-rich stream that will be the feedstock for plastic biopolymers (polyhydroxyalkanoates or PHA) production by bacteria. The bacterial cells already containing PHA suffer a process for the recovery and purification of the polymer that is converted into end products. After polymer recovery, the remaining organic matter will be subjected to anaerobic digestion to produce biogas to generate energy for its use in-situ and fertilisers.

### Mathematical unit models

The equations and assumptions applied to model the unitary operations that compose the process are described below.

A combination of microfiltration (MF), ultrafiltration (UF), and reverse osmosis (RO) has been designed in a cascade configuration. This step has been configured to separate the suspended and dissolved solids (targeted compounds of interest). The equations and assumptions used at the model level are described below ( Corbatón Báguena *et al.*, 2018; Dutta & Nath, 2018; Vinther *et al.*, 2014).

1) Arrhenius model for calculating the viscosity of mixed solutions ( Gao & Li, 2012):

$$\;\ln\mu =\sum X_{i}\ln\mu_{i}\;[1]$$

where,
µ: viscosity of the mixed solution (Pa·s)
X _i_: molar fraction
µ _i_: viscosity of component i (Pa·s)

2) Material balance for suspended solids and solute

$$\;J_{S}=C_{P}\cdot D\cdot J_{V}\;[2]$$

where,
J _S_: suspended solids/solute flux through membrane (mg/h·m ^2^)
C _P_: suspended solids/solute concentration (mg/L)
J _V_: wastewater flux through the membrane (L/h·m ^2^)
D: solids’ diffusivity coefficient

3) Wastewater flux through the membrane (Resistance model)

$$\;J_{V}=\frac{\Delta P_{T}}{R_{m}\cdot \eta }\;[3]$$

where,
J _V_: wastewater flux through the membrane (L/h·m ^2^)
ΔP _T_: Transmembrane pressure (Pa)
R _m_: membrane resistance (m ^-1^)
ƞ: viscosity (Pa·s)

4) Constant-rate filtration

$$\;\frac{t}{V}=\frac{R_{m}\cdot \eta }{\Delta P_{T}\cdot A_{m}}+\frac{\alpha \cdot V^{2}\cdot C_{P}\cdot \eta }{2\Delta P_{T}\cdot A_{m}^{2}}\;[4]$$

where,
t: time (s)
V: Filtered volume (L)
R _m_: membrane resistance (m ^-1^)
ƞ: viscosity (Pa·s)
ΔP _T_: Transmembrane pressure (Pa)
A _m_: Membrane area (m ^2^)
α: specific resistance (m/kg)
C _P_: Suspended solids concentration (mg/L)

5) Carman-Kozeny relationship – Specific resistance

$$\;\alpha =\frac{180(1-\varepsilon )}{\rho \cdot d^{2}\cdot \varepsilon^{3}}\;[5]$$

where,
α: specific resistance (m/kg)
ρ: density (kg/m ^3^)
ε: Fractional pore area
d: solute particle diameter (m)

6) Fractional pore area

$$\;\varepsilon =N\cdot \left(\frac{\pi }{4}\right)\cdot d^{2}\;[6]$$

where,
ε: fractional pore area
N: number of pores/m ^2^
d: solute particle diameter (m)

In order to obtain a simple model based on a mass balance, the following assumptions were considered:

- The filtration processes were set up at constant transmembrane pressure (ΔP _T_).
- The filtration processes were set up at the environmental temperature (T).
- The total resistance in the filtration processes was the membrane resistance (R _m_).
- The diffusivity coefficients of suspended and dissolved solids were fixed independently of temperature (D).

The purpose of the anaerobic fermentation process implemented in AFTERLIFE is the production of volatile fatty acids (VFAs) from the organic matter separated in the steps of filtration and the recovery of value-added compounds.

**Figure 2.**
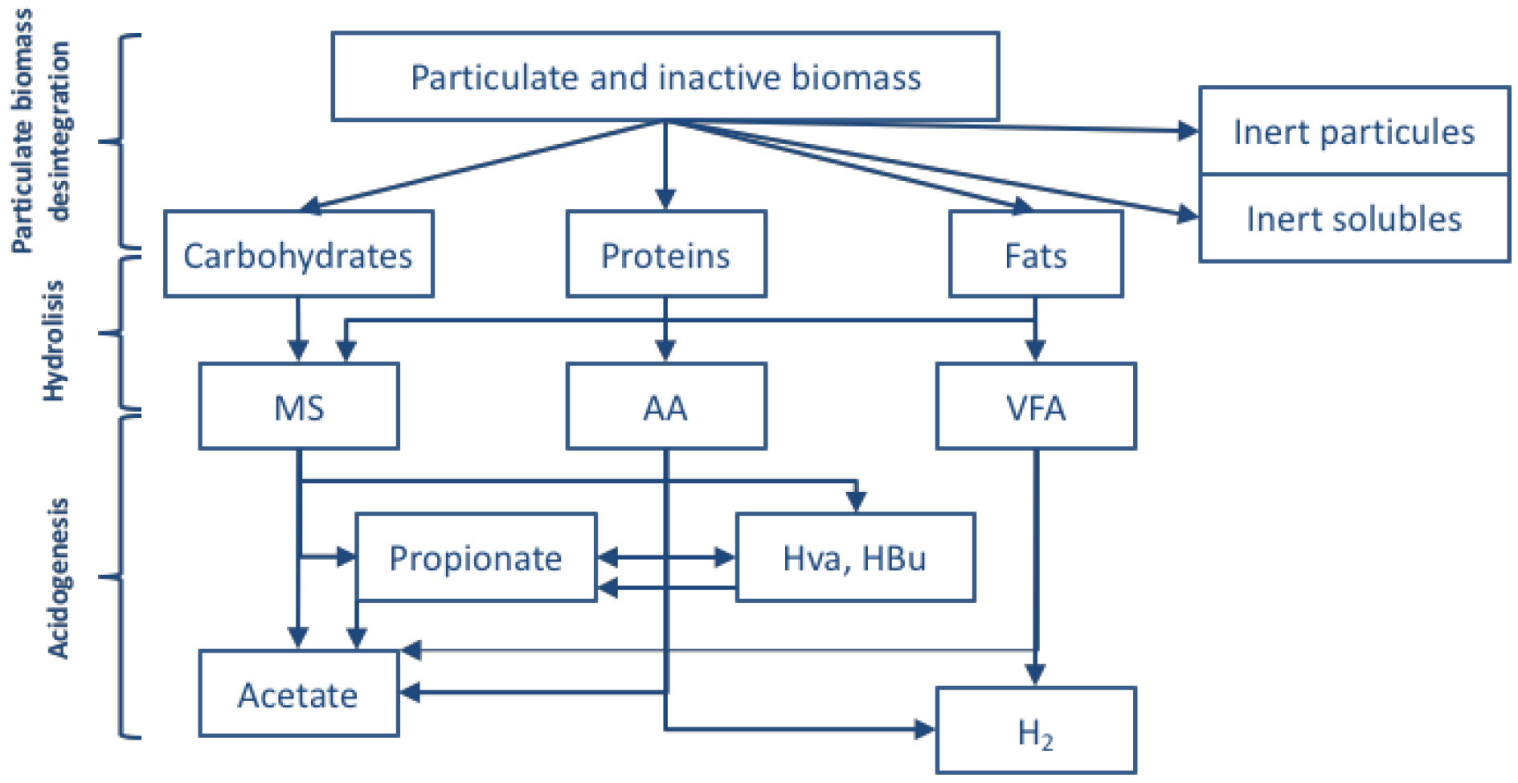
Scheme of processes for the anaerobic fermentation.

The model of this unitary operation is based on the ADM1 model ( Batstone & Vavilin, 2002) ( Figure 2).

Using the ADM1 model as a base, the following equations have been considered to define the anaerobic fermentation process:

1) The disintegration of particulate biomass:

$$\;\frac{dX_{c}}{dt}=\frac{q_{in}}{V_{liq}}\cdot (X_{c,in}-X_{c})-\rho_{c,dis}+\sum_{i}\rho_{i}\;[7]$$

where,
X _c_: concentration of particulate biomass (g DQO· L ^-1^)
q _in_: input flow (L· h ^-1^)
V _liq_: liquid volume in the reactor (L)
ρ _c, dis_: disintegration rate (g DQO· L ^-1^· h ^-1^)
Σρi: the sum of particulate biomass production rate from the cellular decay (g DQO· L ^-1^· h ^-1^)

2) Hydrolytic phase: hydrolysis of carbohydrates, proteins and fats

$$\;\frac{dX_{A}}{dt}=\frac{q_{in}}{V_{liq}}\cdot (X_{A,in}-X_{A})+f_{A,xc}\cdot \rho_{c,dis}-\rho_{A}\;[8]$$

where,
X _A_: concentration of cells able to degrade polysaccharides/lipids/proteins (g DQO· L ^-1^)
q _in_: input flow (L· d ^-1^)
V _liq_: liquid volume in the reactor (L)
f _A, Xc_: compound per unit of particulate biomass (g DQO (A) · g DQO ^-1^(Xc))
ρ _c,dis_: disintegration rate (g DQO · L ^-1^· h ^-1^)
ρ _A_: hydrolysis rate of the compound (g DQO· L ^-1^· h ^-1^)

3) Acidogenic phase: acidogenesis of monosaccharides and aminoacids
Particulate biomass:

$$\;\frac{dX_{B}}{dt}=\frac{q_{in}}{V_{liq}}\cdot (X_{B,in}-X_{B})+Y_{B}\cdot \rho_{B,g}-\rho_{B,dec}\;[9]$$

where,
X _B_: concentration of cells able to degrade monosaccharides/aminoacids (g DQO· L ^-1^)
Y _B_: cellular production per unit of the consumed substrate (g DQO (X)· g DQO ^-1^(X _B_))
ρ _B,g_: cellular substrate consumption rate (g DQO · L ^-1^· h ^-1^)
ρ _B,dec_: cellular decay rate (g DQO · L ^-1^· h ^-1^)

4) Soluble biomass:

$$\frac{dS_{B}}{dt}=\frac{q_{in}}{V_{liq}}\cdot (S_{B,in}-S_{B})+f_{B,A}\cdot \rho_{A}-\rho_{B,g}\;[10]$$

where,
S _B_: monomer concentration (monosaccharides, amino acids, fatty acids)
f _B, A_: monomer production per unit of compound (g DQO (X)· g DQO ^-1^(X _B_))
ρ _A_: hydrolysis rate (g DQO· L ^-1^· h ^-1^)
ρ _B,g_: cellular substrate consumption rate (g DQO · L ^-1^· h ^-1^)

**Figure 3.**
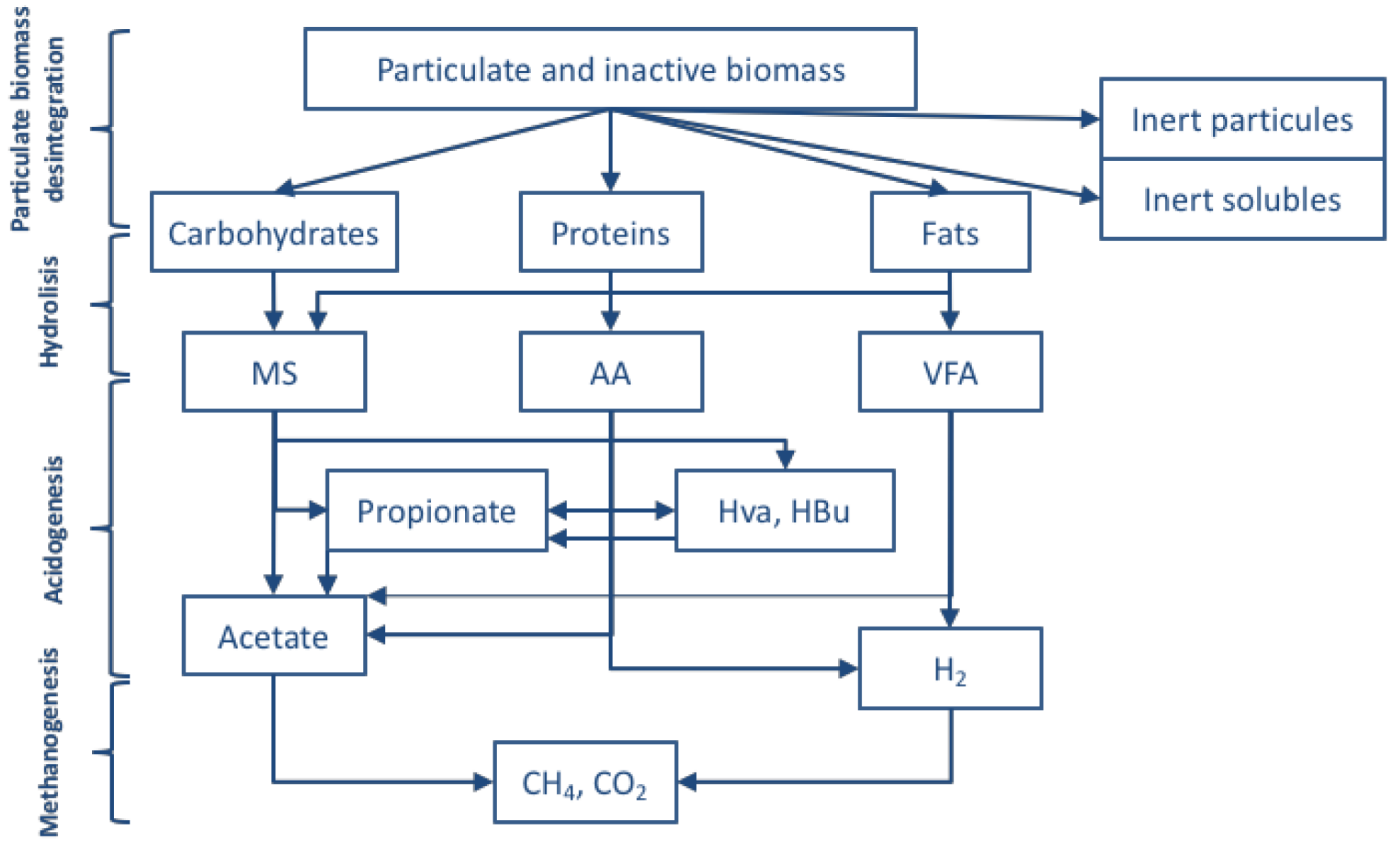
Scheme of processes for the anaerobic digestion.

The anaerobic digestion converts the organic matter into carbon dioxide and methane, which can be used as a source of energy for the process. For the anaerobic digestion ( Figure 3), together with the processes in the anaerobic fermentation, the following phases are considered:

1) Acetogenic phase: acetogenesis of long-chain fatty acids (LCFA), propionate, butyrate and valerate:
Particulate biomass:

$$\frac{dX_{C}}{dt}=\frac{q_{in}}{V_{liq}}\cdot (X_{C,in}-X_{C})+Y_{C}\cdot \rho_{C,g}-\rho_{C,dec}\;[11]$$

where,
X _C_: concentration of cells able to degrade LCFA / propionate / butyrate / valerate
Y _C_: cellular production per unit of the consumed substrate (g DQO (X)· g DQO ^-1^(X _C_))
ρ _C,g_: : cellular substrate consumption rate (g DQO · L ^-1^· h ^-1^)
ρ _C, dec_: cellular decay rate (g DQO· L ^-1^· h ^-1^)
Soluble biomass:

$$\frac{dS_{C}}{dt}=\frac{q_{in}}{V_{liq}}\cdot (S_{C,in}-S_{C})+\sum \left(1-Y_{B}\right)\cdot f_{C,B}\cdot \rho_{B,prod}-\rho_{C,g}\;[12]$$

where,
S _C_: substrate concentration (LCFA, propionate, butyrate, valerate)
f _C, B_: monomer production per unit of compound (g DQO (X)· g DQO ^-1^(X _B_))
Y _B_: cellular production per unit of the consumed substrate (g DQO (X)· g DQO ^-1^(X _B_))
ρ _B,g_: substrate consumption rate (g DQO · L ^-1^· h ^-1^)
ρ _C,g_: cellular substrate consumption rate (g DQO · L ^-1^· h ^-1^)

2) Methanogenic phase: acetoclastic and hydrogenotrophic methanogenesis

$$\frac{dS_{ch4}}{dt}=\frac{q_{in}}{V_{liq}}\cdot (S_{ch4,in}-S_{ch4})+(1-Y_{ac})\cdot \rho_{ac,g}+(1-Y_{h2})\cdot \rho_{h2,g}\;[13]$$

where,
S _Ch4_: methane concentration
Y _ac_: cellular production per unit of consumed acetate (g DQO (X). g DQO ^-1^(X _ac_))
ρ _ac,g_: acetate consumption rate (g DQO · L ^-1^· h ^-1^)
Y _h2_: cellular production per unit of consumed hydrogen (g DQO (X). g DQO ^-1^(X _h2_))
ρ _h2, g_: hydrogen consumption rate (g DQO· L ^-1^· h ^-1^)

The production of the PHA biopolymer uses the VFAs produced during the anaerobic fermentation process as substrate. For this, a feast-famine strategy is applied, that is, the enrichment of the inoculum through cycles of periods of feast and famine. Such a strategy allows for selecting the PHA producing bacteria since the accumulated PHA is a carbon source during the scarcity periods.

For feast and famine periods, the following equations have been considered:

1) Substrate consumption: it is assumed that the consumption of fatty acids follows a Monod kinetics. The mass balance equation will be the following:

$$\frac{dS}{dt}=\frac{q_{in}}{V_{liq}}\cdot (S_{in}-S)-k_{max}\cdot \frac{S}{S+K_{s}}\cdot X\;[14]$$

where,
S: substrate concentration (fatty acid) (g· L ^-1^)
k _max_: maximum substrate consumption rate (g· g (X) ^-1^· h ^-1^)
K _s_: saturation constant (g· L ^-1^)
X: biomass concentration (g· L ^-1^)

2) PHA production: the PHA production depends on the concentration of polymer in the cells and the maximum concentration that could be reached ( Pardelha *et al.*, 2014)

$$\rho_{prod}=\left(1-{\left(\frac{f_{PHA}}{f_{PHA}^{max}}\right)}^{a}\right)\cdot k_{max\cdot }\frac{S}{S+K_{s}}\cdot X\;[15]$$

where,
ρ _prod_: polymer production rate (g· L ^-1^· h ^-1^)
f _PHA_: polymer concentration in cells (g· g (X) ^-1^)
f _PHAmax_: maximum polymer concentration in cells (g· g (X) ^-1^)
a: inhibition factor

3) PHA consumption: the consumption of polymer depends on its concentration in cells and the k constant, which is a function of the feast-famine cycles and of the operation time

$$\rho_{cons}=k\cdot {\left(\frac{X_{o}}{X}\right)}^{1/3}\cdot f_{PHA}^{2/3}\cdot X\;[16]$$

where,
ρ _cons_: polymer consumption rate (g· L ^-1^· h ^-1^)
X _0_: biomass concentration at the beginning of the cycle (g· L ^-1^· h ^-1^)
k: is defined as follows

$$\;k=\frac{2.5}{CL\cdot {\left(\left(\frac{SRT}{CL}\right)-1\right)}^{1/3}}\;[17]$$

where,
C _L_: length of the feast-famine cycle (d ^-1^)
SRT: solids retention time (d ^-1^)
Thus, the material balance for PHA would be:

$$\frac{dPHA}{dt}=\frac{q_{in}}{V_{liq}}\cdot (PHA_{in}-PHA)+\rho_{prod}-\rho_{cons}\;[18]$$

4) Cellular growth: according to Tamis *et al.* (2014), cellular growth is considered the result of the substrate consumption minus the PHA production and that destined for the cellular catabolism

**Figure 4.**
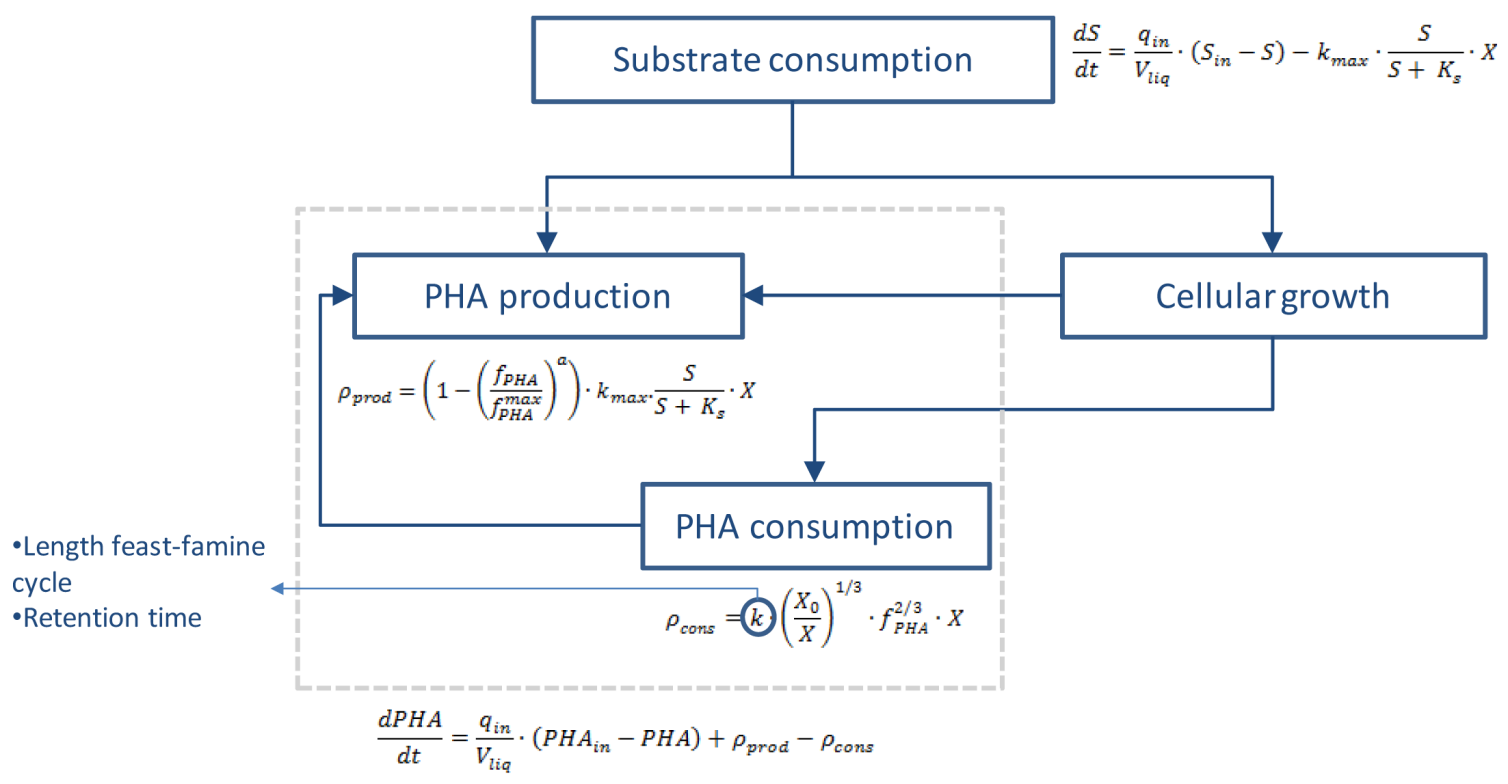
Scheme of processes for the production of PHA.

A scheme of the proposed process to produce PHA is shown in Figure 4.

After culture selection, the polymer is accumulated in a second step where the equations of substrate consumption, cellular growth and PHA production are applicable. Once produced, the polymer should be separated from the cellular biomass. Different strategies are developed during the project for polymer recovery. In this case, the parameters of recovery (percentage of initial polymer recovery after extraction) and purity (percentage of PHA in the recovered solid) are used for modelling the extraction process.

## Implementation

### Process model

The model of the unitary operations has been implemented and coupled in the OpenModelica environment. OpenModelica is an open-source Modelica-based modelling and simulation environment. Modelica is a non-proprietary, object-oriented, equation-based language to conveniently model complex systems.

The coupling of the models in OpenModelica implies the definition of "interfaces" to communicate the information between subsystems. Therefore, the interfaces have been defined considering the variables presented in the different unitary processes.

**Figure 5.**
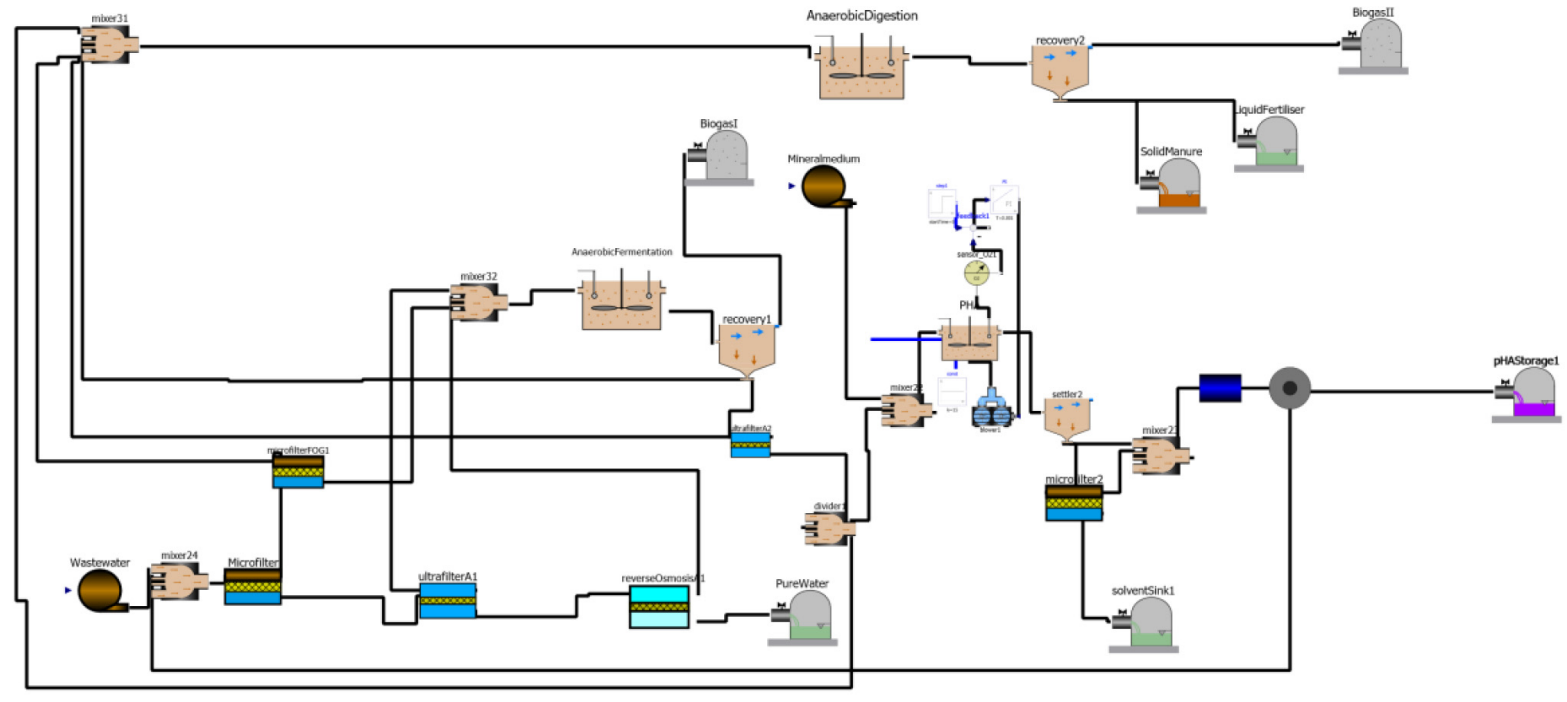
Scheme of processes in OpenModelica (diagram view).

The implemented system has been exported as an image and is shown in Figure 5. Together with the aforementioned models for the unitary operations and the communication interfaces, additional elements have been considered in the implementation. A control system was implemented simulating the air input to the reactor where the PHA production is performed. The air supply to the reactor is controlled to maintain the oxygen concentration at an optimal level for the growth of the microorganisms and the accumulation of PHA. 

### Optimisation of process parameters

**Figure 6.**
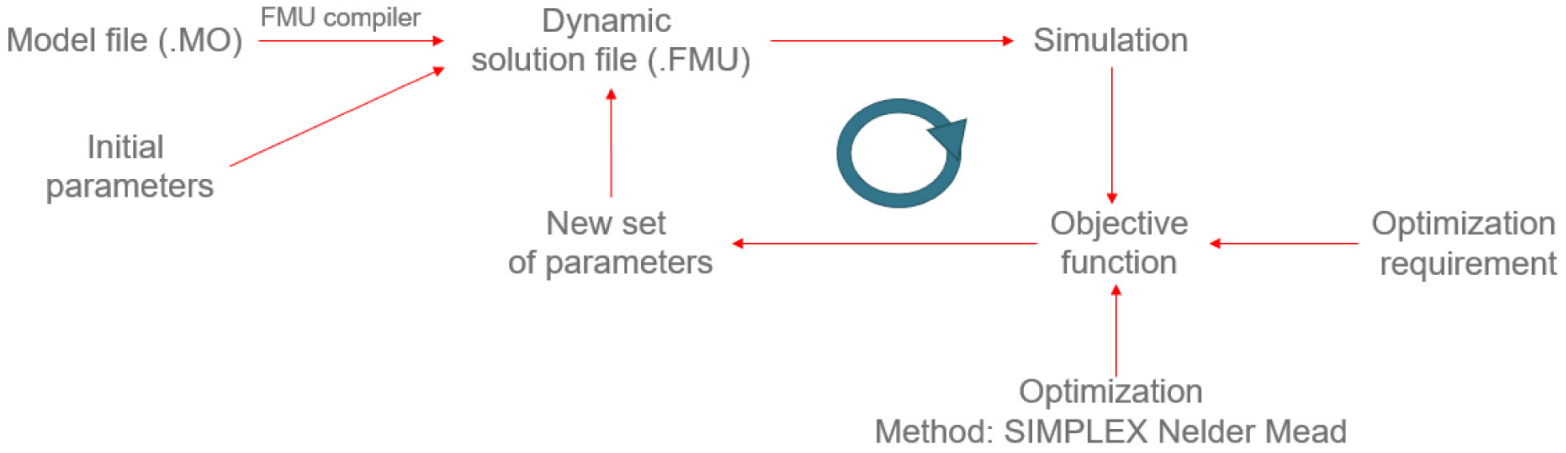
Optimisation methodology.

The following methodology is based on compiling a .MO (Modelica) file to produce an *FMU* (Functional Mock-up Unit) file where the dynamic solution of the model is included. Once an initial set of parameters computes this dynamic simulation, the value of a certain objective function is obtained and compared with the optimisation requirement. If this requirement is not achieved, a new set of parameters is iteratively calculated by an optimisation method up to the objective function value that agrees with the optimisation requirement (see Figure 6).

For the optimisation, the *Python* module *scipy* was used. This module includes several optimisation options, like the Nelder-Mead-Simplex or the Powell methods. However, since the considered model is quite complex, the desired method is the *Nelder-Mead-Simplex* to ensure that an optimum will be found. However, the provided algorithm by *Python* does not allow the definition of boundaries for the *Nelder-Mead-Simplex* method. This fact makes sense since it was developed to evaluate the whole solutions space to find an optimum. Thus, in principle, this method could not be used for our problem since the optimised parameters must be inside a range that ensures the physical sense of the simulation. The routine defined in the file opt2.py was put forward to solve this problem. The idea was that, even when the optimisation method provides values outside the ranges, the evaluated function just considers the limits specified. This correction is done by local variables (a and b) defined inside the function ros. These variables ensure that the model does not use any value out of the fixed bounds. In principle, this solution works, although a certain amount of time will be lost when the solver tries to find an optimum in the discarded ranges.

Concerning optimisation, the objective function must follow some economic and environmental aspects. Net Present Value (NPV) is adopted as an indicator to evaluate the economic performance of the AFTERLIFE process ( Turton *et al.*, 2009). As formulated in [ 19], NPV is determined by capital expenditure (CAPEX), operational expenditure (OPEX), benefits from saleable products (SALE) and discount rate (i). OPEX costs are determined according to chemicals, reagents, and energy costs associated with each year's pilot plant's operation. Additionally, SALES is calculated considering the production yields of value-added products such as PHAs and their average value in the current market. The expected NPV present value minimising decision maker's problem for the AFTERLIFE process is:

$$min-NPV=CAPEX-\sum_{t=1}^{T}\frac{SALE-OPEX}{{\left(1+r\right)}^{t}}\;[19]$$

*s.t.E (NPV _i_) ≥ 0*

*SALES = f(x _1_,y _1_)*

*CAPEX = f(x _1_,y _1_)*

*OPEX = f(x _1_,y _1_)*

where CAPEX, OPEX and SALE denote capital cost, operational cost and the value of the saleable product, respectively, *r* refers to the discount rate, equal to 8%; *t* represents time in years.

The greenhouse gas index (GGI) has been proposed as an indicator concerning environmental aspects. It took information from several sources of GHG emissions from the pilot plant, and CH _4_ emissions were converted into CO _2_-equivalents by calculating a global warming potential (GWP) ( Mannina *et al.*, 2016; Renewables Now, 2017; World Nuclear Association). As formulated in [ 20], it was assumed that part of CH _4_ produced from the anaerobic reactors would be captured for energy recovery or would be flared, and as a result, there would be a small fraction of uncontrollable CH _4_ leaks or inadvertent CH _4_ venting in the system. In the case of CH _4_ capture, CH _4_ was converted into CO _2_ by a chemical CO _2_/CH _4_ equivalent rather than GWP and then classified as a biogenic CO _2_ emission. For this study, the methane capture rate was set to a default factor of 90% according to the IPPC guidelines ( Jigar *et al.*, 2014). The expected greenhouse gas index (GGI) value minimising decision maker's problem for the AFTERLIFE process is:

$$\min\;\mathrm{GGI}=\sum_{t=1}^{T}\left[{\left(G_{i}*GWP\right)}_{i}+(ECi*EF)\right]\;[20]$$

*s.t.GGI > 0*

*G _i_ = f(X _2_,Y _2_)*

*EC _i_ = f(X _2_,Y _2_)*

where *G _i_* is the mass of CO _2_eq, *GWP* is the global warming potential of CO _2_, N _2_O, and CH _4_, *EC _i_* is the energy consumption, and *EF* is the GHG emission factor. *GGI* is influenced by CO _2_eq production from the operation unit *G _i_* and Electricity consumption *EC _i_*

In order to combine both indicators (NPV and GGI), a new NPV corrected with GGI (NPV*) was defined and used for the optimisation:

$$min-NPV^{*}=-NPV+GGI*P_{CO2}\;[21]$$

where *P _co2_* is the allowance price per ton of emitted CO _2_ ( European Commission, 2022).

## Operation

The AFTERLIFE framework ( maloab, 2022) runs using open-source tools ( *OpenModelica* and *Python*), ensuring availability and traceability, enabling interactions with other frameworks, and implementing new process models. It is distributed as a free and open-source tool under Creative Commons Attribution 4.0 International License. Simulations can be performed with Windows and Linux. The minor degree of development of *OpenModelica* for Mac system limits running simulations in it.

### Simulations running

**Figure 7.**
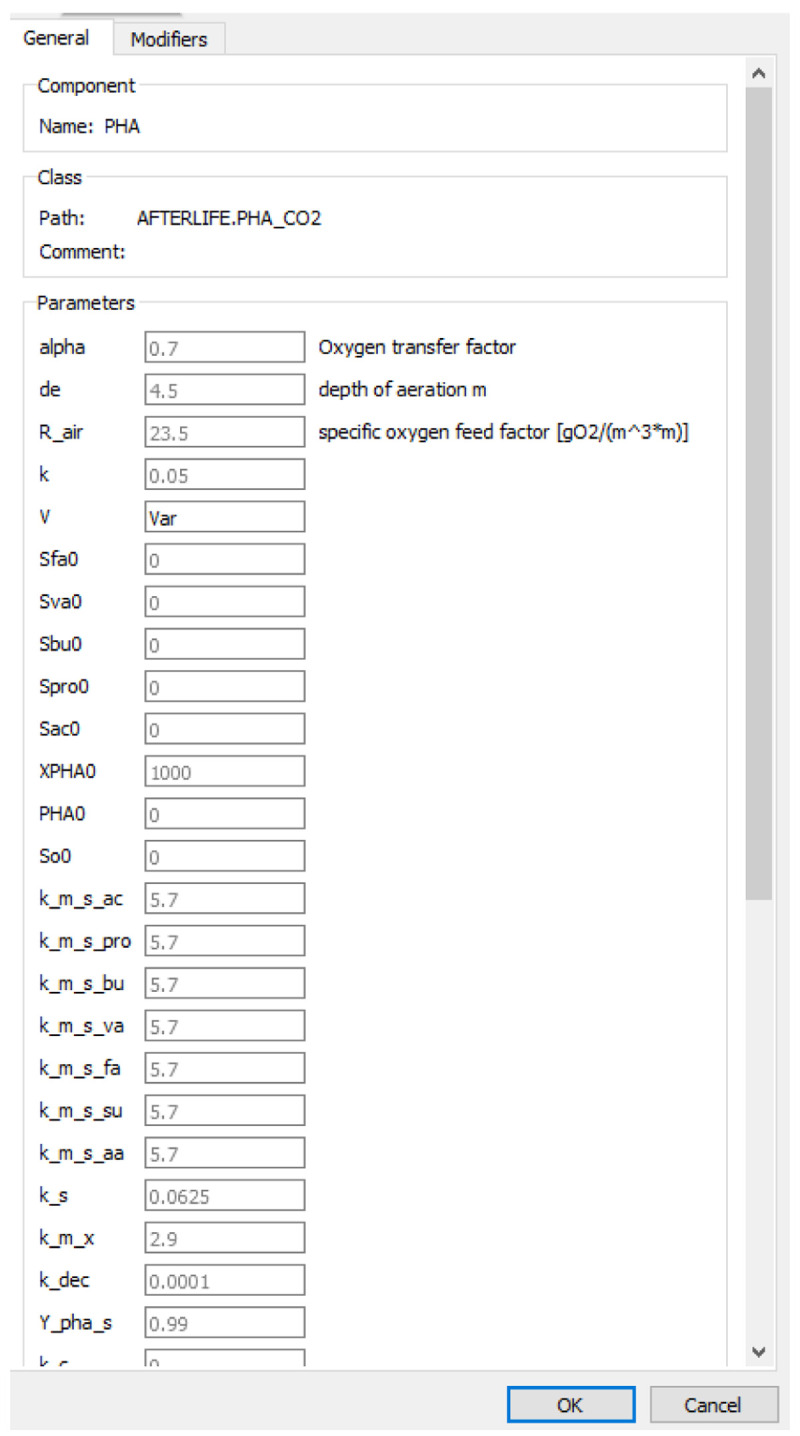
The interface of OpenModelica for identifying the optimisation variables.

First, it is necessary to set the process parameters that can be modified to optimise the overall feasibility of the process. Once selected, they are defined as variables in the python routines. Then, they are linked to the variables in the process model in *OpenModelica* ( Figure 7).

The first step is to connect python with the *OpenModelica* solver (omc.exe). So, a session must be opened using the code in the file Client.py.

Once the session has been defined, an object that contains the considered model is defined. The file model.py does this step. In such a file, the working directory (os.chdir) is changed by the route to a local folder. This modification is required to store make-up files generated during the compilation of the .mo file.

At this point, a session with the omc.exe has been done and also, the model has been stored as a *ModelicaSystem* object. Thus, the following step is to perform a simulation using both. The file simu.py was defined to do so.

This last file has several aspects that should be highlighted. The first one is defining a new value for a parameter in the model. This is done by the sentence: mod.setParameters(Parameter=Value). It should also be noted that this command does not allow the use of phrases (such as Microfilter.Am=0.01). For this reason, every module inside the .mo file must be defined with an input. The following consideration is how to define the simulation conditions. It is done by mod.setSimulationOptions(solver="dassl", startTime=0.0, stepSize=10, stopTime=5000, tolerance=1e-6). Note that, in this case, the provided parameter is the step size and not the number of simulated points. Finally, the simulation is carried out by mod.simulate(). It is also worth highlighting that any number of simulations can be done without a new definition of the ModelicaSystem object.

The selected algorithm was the *Nelder-Mead SIMPLEX* to enhance the possibilities of finding an optimum (a modification was required, see the following sub-section for details). The tool was defined in the file opt2.py. It should be noted that the program developed is based on the definition of a function (ros) where the *OpenModelica* model is evaluated for each value of the optimised parameters provided by the solver.

Since a session and an object are required, this file can only be used if the files Client.py and model.py have been run previously.

### Simulations output

The results from the optimisation can be obtained using the file data.py. The output information is the value of NPV*, the set of optimised process variables and the number of iterations to attempt the optimisation successfully.

## Use case

The process model has been implemented in *OpenModelica*, and simulations have been run considering input wastewater from the fish canning industry. The fish and seafood processing industry generate large quantities of wastewater. The treatment of fish canning wastewater is particularly challenging due to the content of organic matter and salt and the characteristics of the companies, which are widely dispersed and with high seasonal activity. The total soluble and suspended chemical oxygen demand varies meaningfully among factories and fish types. An average characterisation of several wastewaters has been considered and is reflected in Table 1.

**Table 1.** Fish canning effluent characteristics. Source: Cristóvão *et al.* (2015) and Cristóvão *et al.* (2012).

Parameter	Units	Mean	Parameter	Units	Mean
BOD	mg O _2_/L	1546	Conductivity	µS/cm	4.7
COD	mg O _2_/L	1967	Dry residue (105ºC)	mg/L	-
Total Phosphorus	mg/L	31	Sugars	g/100 g	-
Total Kjeldahl Nitrogen	mg/L	211	Amino acids	mg/L	
N-NH _3_	mg/L	3.2	Total		-
N-NO _3_	mg/L	-	Other compounds	mg/L	
pH		6.9	Fat		409
Suspended solids	mg/L	324	Proteins		308.8

The main environmental problems of fishery industries are high water consumption and high organic matter, oil and grease and salt content in their wastewaters. Up to now, the research work has focused on the treatment of these wastewaters to reduce the content in these elements through, e.g., physic-chemical processes (coagulation-flocculation) combined with aerobic biological processes. However, filtration techniques (i.e., reverse osmosis (RO)) have only been tested as polishing steps ( Cristóvão *et al.*, 2012; Cristóvão *et al.*, 2015). The cascade of filtration steps will be an innovative approach that will bring new advantages, such as avoiding the significant production of biological sludges. Moreover, its biodegradability and the content of fat and proteins are interesting to produce VFA.

In a simplified approach, the diffusion coefficients of the simple compounds through the membranes have been set considering the previous knowledge about the typical behaviour of filtration operations. For instance, according to literature ( Hakami *et al.*, 2020; Koyuncu *et al.*, 2015), microfiltration rejects settable and suspended particles larger than 0.1 microns. Thus, the diffusion coefficient was set as zero in the microfiltration step for this fraction. In another case, the diffusion rate has been established as equal to the component concentration in the input stream. On the other hand, the kinetic parameters for VFA, biogas and PHA production have been taken from the existing literature ( Batstone & Vavilin, 2002; Pardelha *et al.*, 2014; Tamis *et al.*, 2014). The parameters in the ADM1 model used for VFA and biogas production were those described by Batstone & Vavilin (2002) and Siegrist *et al.* (2002). Such parameters are considered suitable for wastewater treatment processes in general. Concerning PHA production, the kinetic parameters were taken from Pardelha *et al.* (2014), which proposed a mixed culture process for the conversion of VFA. In this latter model, it should be noted that the culture selection step was not considered since it is expected to make a low contribution from its side to the overall process costs and impacts. Finally, the recovery and purity of PHA in the output of the purification step were set as 100%.

In the current model, the main variables that affect the process are gathered in Table 2. Note that some design variables, like the decanters volume, were not included since they are just related to the processing capacity, but they do not directly affect the performance ( Gawlik, 2018; Renewables Now, 2017 https://renewablesnow.com/news/renewables-produce-337-of-spains-power-in-2017-596136/).

**Table 2.** Main process variables for optimisation.

AFTERLIFE process. Design variables			
ID	Unit	Parameter	Measure
X _AMF_	Microfilter	Membrane Area	m ^2^
X _AUF_	Ultrafilter	Membrane Area	m ^2^
X _ARO_	Reverse Osmosis	Membrane Area	m ^2^
X _VAF_	Anaerobic Fermenter	Volume	m ^3^
X _VAD_	Anaerobic Digester	Volume	m ^3^
X _VAR_	Aerobic Fermenter	Volume	m ^3^

**Figure 8.**
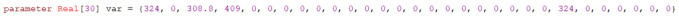
Variable of OpenModelica to introduce wastewater composition.

The composition of the wastewater was used as input in the process model through the variable "var" ( Figure 8).

### Simulation at initial conditions

The selection of design variables to optimise the process is determined by the objective functions described above. Implementing the process to treat the fish processing wastewater with the production of PHA by mixed bacterial culture shows the following variables as non-coupled variables with strong influence in calculating the NPV and GGI values. Table 3 shows the selected variables to optimise, the initial value and the constraint range. These constraint ranges have been determined according to guides on the extensive wastewater treatment processes ( European Commission, 1991). The flow rates of the filtration units have been determined according to the characteristics of the membranes, which determined constraint ranges. The work volume of the reactors has been determined according to the Hydraulic Retention Time (HRT) shown in usual biochemical processes in wastewater treatment plants ( European Commission, 1991) according to the kind of culture and their metabolic routes.

**Table 3.** Initial values and constraint ranges for the selected variables.

Unit	Parameter	Units	Initial capacity	Constraint Range
Microfilter	Membrane Area	m ^2^	0.0014	[0.0009 – 0.002]
Ultrafilter	Membrane Area	m ^2^	0.005	[0.001 – 0.009]
Reverse Osmosis	Membrane Area	m ^2^	0.005	[0.002 – 0.009]
Anaerobic fermenter	Volume	m ^3^	9	[5 – 13]
Anaerobic digester	Volume	m ^3^	20	[10 – 30]
PHA fermenter	Volume	m ^3^	7.5	[4 – 9]

The outcomes of the model are depicted in Table 4, including the final production of PHA, biogas and water and the obtained NPV* and GGI. It was found that the NPV* is positive (that is, the process is profitable with the initial design parameters and the considering inputs and outputs prices). This is mainly possible due to the high PHA price considered (5 €/kg).

**Table 4.** Outputs from the model with the initial parameters.

Model output	Units	Value
Corrected Net Present Value (NPV*)	€	229,865
Greenhouse gasses index (GGI)		12.56
PHA production	g/m ^3^	903.57
Biomethane production	g/m ^3^	428.60
Pure water production	m ^3^/day	9.55

### Simulation at optimised conditions

The model has been optimised by applying the optimisation tool already described in the Method section. The optimised design variables change the operational conditions of the process. Different membrane areas in the filtration units or different work volumes in the reactors suppose changes in the hydraulic retention time (HRT) of the operational units of the process. Table 5 shows the optimal operational conditions. The microfilter, ultrafilter, and reverse osmosis areas keep almost the same in the filtration section. On the other hand, the HRT of the anaerobic digester increases, which means higher production of biogas. The anaerobic and aerobic fermenters show an HRT lower than initial operational conditions decreasing the PHA production of the process. 

**Table 5.** Process parameters before and after optimising.

Unit	Parameter	Units	Initial capacity	Optimised capacity
Microfilter	Membrane Area	m ^2^	0.0014	0.00135
Ultrafilter	Membrane Area	m ^2^	0.005	0.0049
Reverse Osmosis	Membrane Area	m ^2^	0.003	0.003
Anaerobic fermenter	Volume	m ^3^	9	7.6
Anaerobic digester	Volume	m ^3^	20	26.5
PHA fermenter	Volume	m ^3^	7.5	6.15

The model outputs ( Table 6) show that the optimisation favours the production of biomethane in relation to PHA. This way, the NPV would increase, as well as the GGI. This latter is motivated by the increase in biogas production (with 60% of CO _2_) that increases the direct emissions. According to these results, it is possible to reduce the cost of PHA production and, thus, its price in relation to the initial design and increase its competitiveness.

**Table 6.** Outputs from the model with the optimised parameters.

Model output	Units	Value
Corrected Net Present Value (NPV*)	€	236,939
Greenhouse gasses index (GGI)		16.27
PHA production	g/m ^3^	876.25
Biomethane production	g/m ^3^	571.43
Pure water production	m ^3^/day	9.55

**Figure 9.**
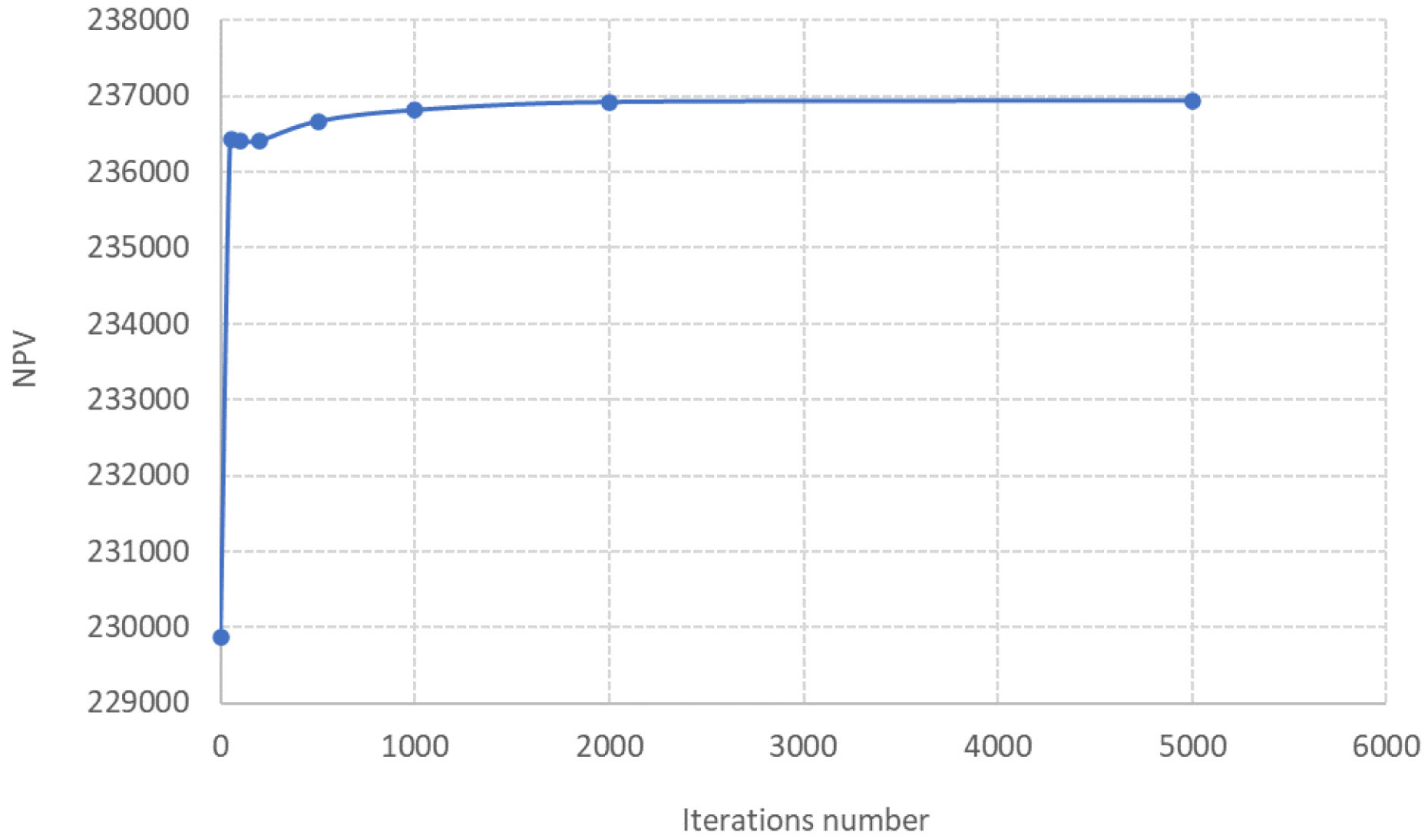
Number of iterations versus NPV (each point is the average of 3 runs).

The number of iterations required to attain the optimised set of values has been evaluated and represented in Figure 9. It can be observed that even at a number as low as 50 iterations, the result is similar to that with a much larger iterations number.

## Discussion and conclusions

The current work presents the AFTERLIFE simulation and optimisation framework and the results of its application for the treatment of food processing wastewater as well as a set of optimised design parameters to consider in the plant design. In the application of multi-objective optimisation, the membrane's area of the filtration step and work volume of the main equipment of the process, i.e., anaerobic digester, anaerobic fermenter and the reactor for PHA production, have been included in the resolution. This way, optimised values for those design parameters have been obtained. Simulations and optimisations trials show positive NPV values, and the set of the optimised design parameters improves this indicator. In the current scenario, the optimisation tool mainly improves the biogas production to reduce the operation cost since the PHA value in the market is low compared to the biogas yield. However, the market scenario is continuously changing, and the current trends show interest in PHA production which could change the market prices. Moreover, the final yields for PHA could be further improved with new bacterial producer strains. This way, the AFTERLIFE process is taking an advantaged position in future market scenarios with a flexible process that treats a reducing cost waste and obtains PHA together with value-added compounds. Additionally, the AFTERLIFE tool allows simulating the performance of the current process scheme for fluctuations in PHA prices, CO _2_ emission allowances, operational conditions and a wide range of industrial wastewater, favouring a wide adoption of the technology.

Three runs have been performed and the results have been averaged to determine each simulation point in Figure 9. The deviation among simulations at the same input conditions was lower than 1% for all of them (Underlying Data AFTERLIFE Model). Thus, the reproducibility of the results can be claimed as high.

Even though experimental works do not verify the present results at lab or pilot scales, knowledge from biochemical processes and process engineering agreed with the findings of this optimisation study. The optimisation can also be repurposed for the parametrisation of the models with real experimental data. The main adaptation is that the objective function should be replaced by a quadratic difference between the experimental and the simulated values.

Although the application of the current framework requires reliable mathematical models for the different process steps, it can be affirmed that such models are broadly available in the literature. ADM1 or PHA production models are a probe of this. Moreover, plenty of studies go from simple unstructured models to more rigour metabolic network models.

Besides, as the framework is generic, it can be applied to evaluate other processing schemes by including new units or modifying the current ones. Moreover, it was found that a low number of iterations is required to attempt a good optimisation performance, which reduces the time and computational demand of the framework. Finally, the comparison of the same process with alternative optimisation frameworks based on stochastic techniques will be extremely interesting to assess the performance of both approaches jointly.

## Data availability

### Underlying data

Zenodo: Underlying data AFTERLIFE model. https://doi.org/10.5281/zenodo.6543225 ( Lopez, 2022)

This project contains the following extended data:

- Extended data AFTERLIFE model.xlsx [Set of simulation results using AFTERLIFE framework at different number of iterations]

Data are available under the terms of the Creative Commons Attribution 4.0 International license (CC-BY 4.0).

## Software availability

Source code available from: https://github.com/maloab/AFTERLIFE-H2020-project

Archived source code at time of publication: https://www.doi.org/10.5281/zenodo.6424235 ( maloab, 2022)

License: Creative Commons Attribution 4.0 International License
