# Supplementary material for: Modelling and mathematical optimisation of wastewater treatment in food industries
Source: Open Res Eur. 2022 May 19;2:62. [Version 1] doi: 10.12688/openreseurope.14737.1 (PMC10446076; doi:10.12688/openreseurope.14737.1)
Supplement: Peer review history

# Peer review: Reviewer response for version 1

**DOI:** 10.21956/openreseurope.15919.r31378

**Published:** 2023-05-22

**Authors:** Serkan Eker

**Affiliations:** 1 Dokuz Eylul University, İzmir, Turkey

The paper presents the work conducted within the AFTERLIFE project, which emphasizes bioprocess modeling and optimization through computational tools. The project's goal is to create a flexible, cost-effective, and resource-efficient process for recovering and valorizing significant fractions from wastewater, adopting a zero-waste and circular economy approach. The paper highlights the development and implementation of computational tools for modeling and optimizing the process design.

 The process model and optimization methodology were employed to simulate wastewater treatment from the fish processing industry. The development and implementation of computational tools for process design modeling and optimization are detailed in the paper. A modeling-based optimization framework has been established, and the applied optimization approach is computationally efficient and systematically applicable to processes.  The optimization tool's performance is assessed based on the simulation results, providing sufficient information to understand the expected output datasets and any results generated using the tool.

 Since no distinct software programs are utilized, it may be easily adapted to other projects. (But you'll need the assistance of knowledgeable end-users for this.) The article is suitable for the target audience of researchers and professionals in the fields of wastewater treatment and bioprocess optimization.

 Small changes are needed:

- high-pure extracts- high-purity extracts
- physic-chemical processes - physico-*chemical processes
- biological sludges- biological sludge
- In Fig 5. Process diagram view. It is quite difficult to read the name of the units in the diagram (ppt). I checked to this diagram from the OpenModelica. 

Is the rationale for developing the new software tool clearly explained?

Yes

Is the description of the software tool technically sound?

Yes

Are sufficient details of the code, methods and analysis (if applicable) provided to allow replication of the software development and its use by others?

Yes

Is sufficient information provided to allow interpretation of the expected output datasets and any results generated using the tool?

Yes

Reviewer Expertise:

Environmental engineering, biological treatment: modeling and optimization, wastewater treatment systems; bioconversion of waste, renewable energy, biofuel production

I confirm that I have read this submission and believe that I have an appropriate level of expertise to confirm that it is of an acceptable scientific standard.

## References

[1] : Modeling and optimization of wastewater treatment processes.2021; 10.1016/B978-0-12-821009-3.00006-3 373-396 10.1016/B978-0-12-821009-3.00006-3

[2] : Modeling the impact of food wastes on wastewater treatment plants. *J Environ Manage* .2019;237: 10.1016/j.jenvman.2019.02.065 344-358 10.1016/j.jenvman.2019.02.065 30818237

# Peer review: Reviewer response for version 1

**DOI:** 10.21956/openreseurope.15919.r30099

**Published:** 2022-10-21

**Authors:** Carmen Zaharia

**Affiliations:** 1 Faculty of Chemical Engineering and Environmental Protection; Department of Environmental Engineering and Management, “Gheorghe Asachi” Technical University of Iasi, Iasi, Romania

The manuscript subject is challenging, actual, and important in developing flexible, cost-efficient processes as well as good practices for waste recovery and valorization, especially in the food industry but in a controlled, or computer-assisted manner in which the modelling and mathematical optimization of bioprocesses have a significant role. It is a study that considered the wastewater treatment in the fish processing industry and after treatment and valorization of some separated fractions by adequate bioprocesses as value-added products, thus minimizing the waste quantity and also recovery of useful compounds as it is recommended by the circular economy.

 I carefully read the manuscript and I like it, being clear, logical, and well-written. Moreover, the treated subject is a recognized concern of mankind which prioritizes the progress and developing requirements, i.e. low-cost and facile way of separating different fractions from industrial wastewaters (filtrated solids with numerous organics, nutrients such as N, P, metal microelements, etc.) by efficient membrane processes, and after their treatment by bioprocesses as well as wet processes, such as digestion/fermentation for useful products obtaining/preparation (e.g., extracts, metabolites, etc.). Therefore, the total separation of solids in sewage was proposed to be achieved through a cascade of membrane filtration units and after the concentrates were treated to obtain high pure extracts and metabolites or converted into value-added biopolymers (polyhydroxyalkanoates). The final filtration outflow is an ultra-pure water stream that can be recycled. This complex technological treatment system was discussed and named the AFTERLIFE process and was designed and optimized to always get an adequate performance and reduced costs in connection with maximizing recoveries from the wastewater treatment systems. In this case, there were utilized efficient and corresponding computational tools to design and optimize a wastewater treatment process from the fish processing industry. The performance of simulation, modelling, and mathematical optimization tools was evaluated and validated and their beneficial effects were underlined.

 Regarding the question, " *Is sufficient information provided to allow interpretation of the expected output datasets and any results generated using the tool?*", for non-specialized readers, the answer is 'Partly', as the understanding of statistics and model interpretation is not an easy one to process. But for specialized readers, the answer is 'Yes' as there are enough data which can be processed by them to see if the same results are obtained, and thus to understand that some approaches were considered for data appreciation, interpretation, and validation.

 I like this manuscript and I consider that, after a few minor corrections, this manuscript can be accepted for indexing.

 Minor corrections required:

1. A few English grammar and spelling corrections are required.
2. In Figure 3, page 7, correction of the term as ‘hydrolysis’.
3. I recommend using the terminology of ‘physical-chemical’ instead of ’physic-chemical’, page 12, line 5, second column.
4. In Table 1, page 12 must be inserted the measurement units for two elements (as mg/L).

These are some of the minor corrections required for the improvement of the manuscript quality.

Is the rationale for developing the new software tool clearly explained?

Yes

Is the description of the software tool technically sound?

Yes

Are sufficient details of the code, methods and analysis (if applicable) provided to allow replication of the software development and its use by others?

Yes

Is sufficient information provided to allow interpretation of the expected output datasets and any results generated using the tool?

Yes

Reviewer Expertise:

Environmental engineering and management; chemical engineering; simulation, modelling and optimization of water/wastewater treatment technological processes; energy and environment; waste treatment and valorization, environmental chemistry, among others.

I confirm that I have read this submission and believe that I have an appropriate level of expertise to confirm that it is of an acceptable scientific standard.
